# Use and Abuse of Hypnotics in Insomnia

**Published:** 1899-02

**Authors:** 


### Use and Abuse of Hypnotics in Insomnia.

According to the *Post-Graduate* (Vol. 13, No. 5) the use of hypnotics in the treatment of insomnia is simply the use of symptom-remedies; insomnia is a symptom, not a cause of disease nor a disease. Sleep is essential to the welfare of the organism in the same sense that food is. Deprivation of one or the other causes death in about the same period of time.

The use of hypnotics, therefore, should be temporary while the underlying cause of the insomnia is being removed or palliated. Nor, indeed, is it well at the outset to employ hypnotics without trial of other measures. Aside from the removal of somatic causes for sleeplessness, various general methods may be employed. One of the best is a bath at 104° F. for five minutes. The general cutaneous vascular dilatation, increased by rubbing with a coarse towel, is frequently followed by a good night's rest. Warm liquid food, as a glass of hot milk, a bowl of soup, will often give satisfactory results. In fact, some of the hypnotics which, on account of their insolubility, must be given in considerable quantities of hot liquids, owe not a little of their reputation to the vehicle in which they are administered. In debilitated individuals, a glass of stout or whisky in hot water (hot Scotch) may work wonders. In tired subjects, strychnine sulphate in moderate dose acts as a hypnotic, not because it makes a too tired individual just tired enough to sleep, as a distinguished professor of medicine would have it, but because strychnine dilates the arterioles. Sometimes stimulation of the emunctories, as by sodium sulphate, again in hot water, taken at night will be followed by sleep, particularly in gouty subjects, not because it is hypnotic, but on account of its action on the liver, intestines and kidneys. Methods which relieve pain—position, topical applications—are hypnotic.

Sleep is accompanied by cerebral anemia and systematic cutaneous vascular dilatation. Any method which produces these effects will tend to the production of sleep. When these all fail, and after they do, hypnotics must be resorted to. The safest only should be chosen; they are chloralamid, pellatine, paraldehyde and trional. The danger of hypnotics is immediate (death) or remote (interference with nutrition). The possibility of habit is always to be borne in mind. According to the article, druggists are responsible for a large share of the abuse of hypnotics. Many openly prescribe hypnotics in doses far exceeding those considered safe, and, further, repeat prescriptions containing hypnotic drugs, even when the prescription directly forbids this. In England, sulphonal is sold as openly and carelessly as are the ordinary necessities of life. With equal ease coffee can be purchased for breakfast and sulphonal for bedtime. According to the writer, the same is true in this country. The only remedy lies with the physician. Let him study his materia medica, learn his therapeutics, and apply intelligently what he has learned. Then, and only then, may he get the best results with the fewest disadvantageous symptoms, do the most for his patients, and, after all, rest with a consciousness of duty well performed.—*American Medico-Surgical Bulletin.*

THE Lyon *Medical* says that washing the hands with orange juice water after using iodoform dispels the unpleasant smell.

